# New dinosaur (Theropoda, *stem*-Averostra) from the earliest Jurassic of the La Quinta formation, Venezuelan Andes

**DOI:** 10.1098/rsos.140184

**Published:** 2014-10-08

**Authors:** Max C. Langer, Ascanio D. Rincón, Jahandar Ramezani, Andrés Solórzano, Oliver W. M. Rauhut

**Affiliations:** 1 Laboratório de Paleontologia de Ribeirão Preto, FFCLRP, Universidade de São Paulo, Avenida Bandeirantes 3900, 14040-901, Ribeirão Preto-SP, Brazil; 2 Laboratorio de Paleontología, Centro de Ecología, Instituto Venezolano de Investigaciones Científcas (IVIC), Carretera Panamericana Km 11, 1020-A Caracas, Venezuela; 3 Department of Earth, Atmospheric and Planetary Sciences, Massachusetts Institute of Technology, 77 Massachusetts Avenue, Cambridge, MA, USA; 4 SNSB, Bayerische Staatssammlung für Paläontologie und Geologie and Department of Earth and Environmental Sciences, Ludwig-Maximilians-University, Richard-Wagner-Strasse 10, Munich, Germany

**Keywords:** Averostra, Dinosauria, Early Jurassic, U–Pb geochronology

## Abstract

Dinosaur skeletal remains are almost unknown from northern South America. One of the few exceptions comes from a small outcrop in the northernmost extension of the Andes, along the western border of Venezuela, where strata of the La Quinta Formation have yielded the ornithischian *Laquintasaura venezuelae* and other dinosaur remains. Here, we report isolated bones (ischium and tibia) of a small new theropod, *Tachiraptor admirabilis* gen. et sp. nov., which differs from all previously known members of the group by an unique suite of features of its tibial articulations. Comparative/phylogenetic studies place the new form as the sister taxon to Averostra, a theropod group that is known primarily from the Middle Jurassic onwards. A new U–Pb zircon date (isotope dilution thermal-ionization mass spectrometry; ID-TIMS method) from the bone bed matrix suggests an earliest Jurassic maximum age for the La Quinta Formation. A dispersal–vicariance analysis suggests that such a stratigraphic gap is more likely to be filled by new records from north and central Pangaea than from southern areas. Indeed, our data show that the sampled summer-wet equatorial belt, which yielded the new taxon, played a pivotal role in theropod evolution across the Triassic–Jurassic boundary.

## 2. Introduction

Averostra, the major-group of theropod dinosaurs that includes Tetanurae and Ceratosauria [1,2] is well known from the Middle Jurassic onwards [3,4], but closely related forms have been recovered from various Late Triassic–Early Jurassic deposits around the world [5–10]. Many of these taxa have controversial phylogenetic positions [4,11,12], with contested referrals to Coelophysoidea [1] or to the ‘*Dilophosaurus* clade’ [9], hampering the identification of averostran diagnostic traits, as well as the inference of their ancestral biogeographic range. However, the latter challenge may be overcome by the discovery of new fossils, especially from poorly sampled areas.

**Figure 1.**
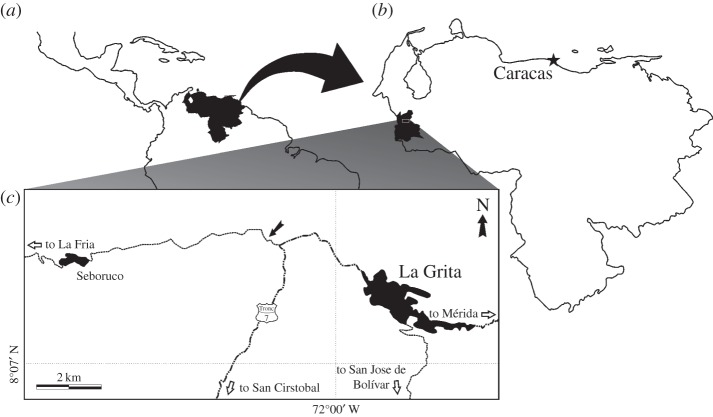
Maps of (*a*) Venezuela within northern South and Central America, (*b*) Táchira State within Venezuela and (*c*) La Grita area indicating the location of the type locality of *Tachiraptor admirabilis* (black arrow). Dash-dotted lines denote main roads; thin dotted lines, paved secondary roads.

Dinosaur skeletal remains are almost unknown in some Gondwanan areas, such as the northern part of South America [13]. The latter has yielded mostly fragmentary remains from Brazil, Colombia and Venezuela [14–16]. The best known of these are found in a single, relatively small outcrop of the La Quinta Formation (figure 1) in the area of the Mérida Mountains, the northernmost extension of the Andes, at the western border of Venezuela. Its interbedded tuff and siltstone intervals have yielded fish remains, isolated teeth of a carnivorous archosaur, and isolated bones and teeth of the small ornithischian *Laquintasaura venezuelae* [17–20].

The La Quinta Formation consists mainly of continental red beds and volcanic rocks deposited in an extensional tectonic setting associated with the Mesozoic breakup of Pangaea and opening of the Atlantic Ocean [21–24]. In its type locality, near the town of La Grita, the La Quinta Formation is over 1600 m thick and exceptionally tuffaceous. It lies unconformably atop the low-grade metamorphic rocks of the Mucuchachi Formation of middle Carboniferous to Permian age, and is covered, via a disconformity or transitional interval, by the Lower Cretaceous Rio Negro Formation [25]. The type-section can be divided into three distinct intervals [25], the middle of which comprises 840 m of tuffs, interbedded with siltstones, sandstones and local layers of limestone. Its depositional environment has been interpreted as an alluvial plain, under a seasonally arid and humid tropical climate [25]. Sedimentation was predominantly fluvial, locally lacustrine, disrupted by widespread pyroclastic input. The mafic lavas and shallow intrusions that characterize the La Quinta Formation in other areas (e.g. Sierra de Perijá [21]) are absent from the type locality.

The age of the La Quinta Formation has been debated in the literature [20]. In the type area (Mérida Andes), siltstones, shales and limestones of the middle interval contain plant remains, ostracods, conchostracans, palynomorphs and fish teeth, most of which are poorly preserved. The fossil plants suggest an age ranging from Early Jurassic to Early Cretaceous [26]; whereas palynomomorphs indicate an older Late Triassic to Middle-Late Jurassic age [25]. In addition, the record of a dinosaur ilium with a not fully perforated acetabulum suggests a Late Triassic to Early Jurassic age [19,20]. In the Perijá Andes, the palaeobotanical association of the La Quinta Formation suggests a Middle Jurassic age [26,27], and abundant estherid conchostracans range in age from Late Triassic/Early Jurassic, in the lower part of the unit, to Late Jurassic/Early Cretaceous, in its upper part [21].

Past geochronological studies of the La Quinta Formation have produced equivocal results. A U–Pb zircon date (bulk multi-grain zircon analyses) of 229±15 Ma from the La Grita dacitic tuff at the base of the La Quinta Formation in the Mérida Andes [28], suggested a Late Triassic episode of volcanic activity. Biotite and whole-rock K–Ar dates reported from the basal tuff were, however, much younger at 149±10 Ma and 122.5±7.7 Ma, respectively [25,29], probably owing to thermal perturbation of the K–Ar system at a younger date. Reported U–Pb, Rb–Sr and K–Ar dates from volcanic rocks of the La Quinta Formation in the Perijá Andes [21] range from Middle to Late Jurassic (approx. 170–140 Myr ago). Together, the radioisotopic dates and scant fossil evidence suggest that the deposition of the La Quinta Formation started in the Late Triassic.

During the last few years, several field investigations have been conducted around the type-section of the La Quinta Formation, allowing the collection of new dinosaur material. Here, two theropod bones (tibia and ischium) are reported. They represent a new member of that clade, increasing the depauperate dinosaur record of northern South America. Furthermore, new U–Pb zircon geochronology (chemical abrasion thermal-ionization mass spectrometry; CA-TIMS method) from the fossil-bearing, tuffaceous bed provides more robust constrains on the age of the La Quinta Formation and its important biota.

## 3. U–Pb geochronology

### 3.1 Sample and methods

Zircons were separated from a sample of relatively uniform, grey, tuffaceous siltstone that enclosed the theropod fossils by standard heavy mineral separation techniques using high-density liquids. Seven single zircons were selected based on grain morphology and analysed by the U–Pb isotope dilution thermal-ionization mass spectrometry (ID-TIMS) technique following the procedures described in [30]. All zircons were pre-treated by a CA-TIMS method modified after [31] to mitigate the effects of radiation-induced Pb loss, and spiked with the EARTHTIME ET535 mixed ^205^Pb–^233^U–^235^U tracer prior to dissolution and analysis. Data reduction including date calculation and propagation of uncertainties was carried out using computer applications and algorithms of [32,33]. Complete U–Pb data appear in the electronic supplementary material, table S1.

In the case of tuffaceous (volcaniclastic) sedimentary deposits, sample date is calculated based on the weighted mean ^206^Pb/^238^U date of a coherent cluster of the youngest zircon analyses from the sample and is interpreted as the maximum age of deposition. Uncertainties are reported at 95% confidence level and follow the notation ±*X*/*Y* /*Z* Ma, where *X* is the internal (analytical) uncertainty in the absence of all external errors, *Y* incorporates the U–Pb tracer calibration error and *Z* includes the latter as well as the decay constant errors of [34]. Complete uncertainties (*Z*) are necessary for comparison between age data from different isotopic chronometers (e.g. U–Pb versus ^40^Ar/^39^Ar).

### 3.2 Results and age constraints

**Figure 2.**
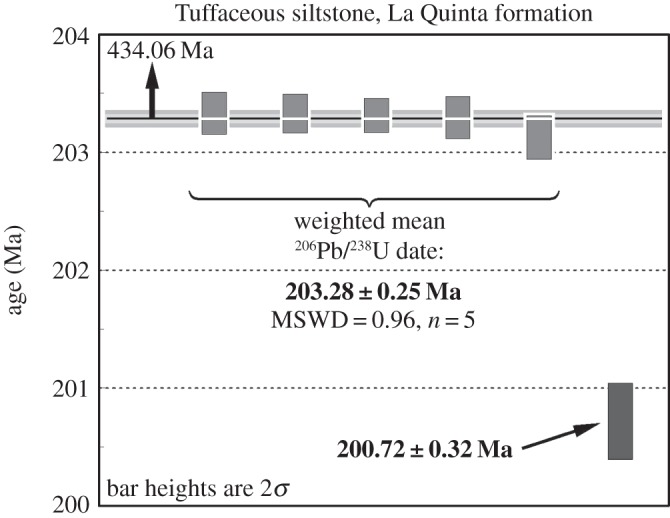
Date distribution plot of analysed zircons of this study. Bar heights are proportional to 2*σ* analytical uncertainty of individual analyses; solid bars are analyses used in age calculation. Horizontal lines signify calculated sample dates and the width of the shaded band represents internal uncertainty in weighted mean at a 95% confidence level. Arrow points to additional analysis plotting outside the diagram. Reported date incorporates external sources of uncertainty. See the electronic supplementary material, table S1 for complete analytical data and text for details of date uncertainties.

Selected zircons for analysis (see the electronic supplementary material, figure S1) were sharply faceted prisms with visible glass (melt) inclusions along their ‘*c*’ axes and with no detectable evidence of abrasion or rounding. Five of the analysed zircons form a coherent cluster with a weighted mean ^206^Pb/^238^U date of 203.281±0.075/0.12/0.25 Ma and a mean square of weighted deviates (MSWDs) of 0.96 (figure 2). One significantly older outlier (z6) at *ca* 434 Ma is interpreted as detrital, whereas another analysis (z5) at 200.72±0.32/0.34/0.40 Ma is younger outside the uncertainty from the main cluster. The latter could be the result of persistent Pb loss or possibly reflect a younger, underrepresented, population of zircons (see below).

A parallel U–Pb geochronologic (CA-TIMS) study of the La Quinta Formation bone bed near La Grita [20] has identified a spectrum of zircon dates, though with higher uncertainties, fairly comparable to those reported here (see the electronic supplementary material, figure S1). These include a main population of analyses clustered around *ca* 203.48 Ma, as well as two distinguishably younger zircons at 200.98±0.62 Ma and 200.6±1.4 Ma (2*σ* errors [20]). The latter two overlap within uncertainty with our single youngest zircon analysis, providing further evidence in support of a younger zircon population present in the sample. Our measured date of 200.72±0.32 Ma thus represents a closer estimate for the maximum age of deposition of the fossil-bearing bed than the above calculated weighed mean date.

The most recent calibration of the terminal Triassic timescale based on U–Pb geochronology of ammonite-bearing marine strata of the Pucara Basin in northern Peru places the Norian-Rhaetian and the Rhaetian-Hettangian (Triassic–Jurassic) boundaries at 205.50±0.35 Ma and 201.36±0.17 Ma, respectively [35]. Accordingly, the fossiliferous bed of the La Quinta Formation with an estimated age equal to, or younger than 200.72±0.32 Ma could have been deposited as early as 150 kyr after the start of the Jurassic. Since our U–Pb age constraint for the bed is only a maximum estimate (see above), it is possible that the true depositional age of the bed is appreciably younger than our measured date. The magnitude of this possible age bias cannot be reliably quantified unless additional, closely spaced, tuffaceous samples from the same stratigraphic section are dated [30].

## 4. Systematic palaeontology

Theropoda Marsh 1881 *sensu* [36]

Neotheropoda Bakker 1986 *sensu* [37]

*stem*-Averostra Paul 2002 *sensu* [1]

*Tachiraptor admirabilis* new genus and species

### 4.1 Etymology

The generic name derives from Táchira, the Venezuelan state where the fossil was found, and raptor (Latin for thief), in reference to the probable predatory habits of the animal. The specific epithet honours Simon Bolivar's ‘Admirable Campaign’, in which La Grita, the town where the type locality is located, played a strategic role.

### 4.2 Holotype and referred material

**Figure 3.**
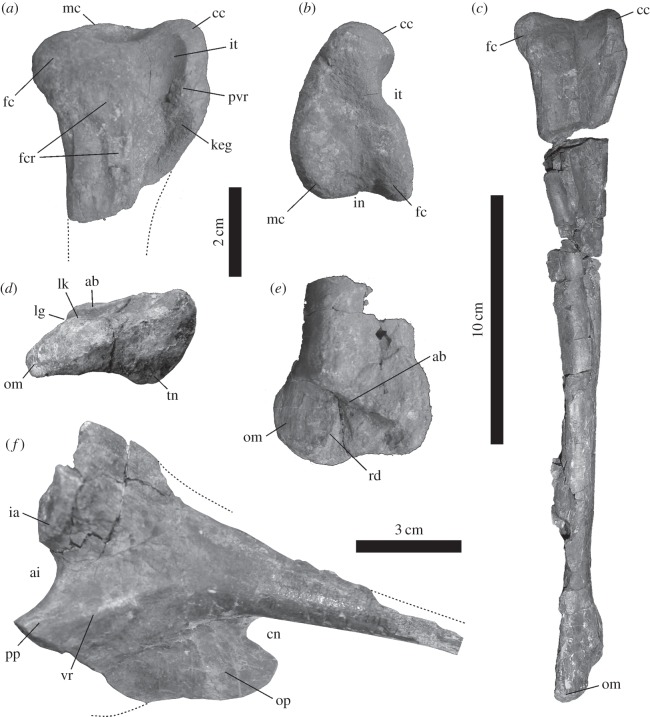
*Tachiraptor admirabilis* gen et sp. nov. Holotype right tibia (IVIC-P-2867) in (*a*) lateral (proximal portion), (*b*) proximal, (*c*) lateral, (*d*) distal and (*e*) cranial (distal portion) views. Referred left ischium (IVIC-P-2868) in (*f*) lateral view. Abbreviations: ab, astragalar buttress; cc, cnemial crest; ai, cranial emargination; cn, caudal notch; fc, fibular condyle; fcr, fibular crest; ia, iliac articulation; in, intercondylar notch; it, incisura tibialis; keg, ‘knee extensor groove’; lg, longitudinal groove; lk, lateral kink; mc, medial condyle; om, outer malleolus; op, obturator plate; pp, pubic peduncle; pvr, ‘postero-ventral ridge’; rd, ridge; tn, tibial notch; vr, ventral ridge.

Holotype: IVIC-P-2867 (see institutional abbreviations in the electronic supplementary material): nearly complete right tibia (figure 3*a*–*e*). Referred material: IVIC-P-2868: proximal left ischium (figure 3*f*) found in the same spot as the type-material.

### 4.3 Type locality and horizon

Greenish siltstone at the lower third of the ‘middle interval’ of the La Quinta Formation [25], exposed at a secondary road (72^°^01^′^06.60 W, 08^°^09^′^03.47 N) next to the north of the type-section and about 4 km northwest of the town of La Grita, Jáuregui municipality, Táchira State, Venezuela (figure 1).

### 4.4 Diagnosis

Distinguished from all other theropods by the following unique combination of characters (possible autamorphy among early theropods marked with an asterisk): caudolateral corner of the fibular condyle forms a sharp angle in proximal view and extends slightly more caudally than the medial condyle*; distal articulation of the tibia more than 50% broader transversely than craniocaudally (despite slight postdepositional deformation); astragalar buttress occupies between one-third and one-quarter of the craniocaudal depth of the distal surface of the bone, extending obliquely across the cranial surface of the distal part of the tibia at an angle of approximately 35^°^ to the distal margin, and flexing proximally at the lateral 20% of the transverse width of the distal shaft; line connecting the outer and inner tibial malleoli in cranial view forms an angle of *ca* 80^°^ to the long axis of the bone.

### 4.5 Comments

Regardless of the peculiar morphology of the distal part of its tibia, the uniqueness and inclusivity of *T. admirabilis* is inferred for topotypical reasons. Its holotype tibia and referred ischium were found at the same locality, have concordant phylogenetic signals, equivalent relative sizes, and are the only unequivocal theropod remains recovered from the La Quinta Formation after more than 20 years of research.

## 5. Description

Based on the proportions of its holotype right tibia (IVIC-P-2867), *T. admirabilis* was a small theropod, with an estimated body length slightly over 1.5 m. The approximately 25 cm long tibia is on average 20 mm broad across the mid-shaft; these skeletal proportions are shared by other basal theropods of corresponding size such as ‘*Syntarsus*’ *kayentakatae* [38] and the ‘Petrified Forest’ coelophysoid [39].

The tibia is nearly complete (figure 3*a*–*e*), but fractured in several positions. The proximal articulation is subtriangular, with subequal medial and fibular condyles (the latter extending slightly more caudally) separated by a subtle caudal notch (‘in’ in figure 3*b*). The caudomedial corner of the medial condyle is rounded, whereas the laterocaudal corner of the fibular condyle forms a sharp angle of approximately 75–80^°^. The articular surface is excavated at its centre, leading to three elongated depressions that separate the cnemial crest and the caudal condyles. The cnemial crest is notably expanded cranially from the tibial shaft and slightly proximally relative to the fibular condyle, although level with the medial condyle. Measured from the caudal margin of the incisura tibialis, the crest occupies about 50% of the craniocaudal length of the articulation. The incisura tibialis is well developed, but shallow. It separates the fibular condyle from a proximodistally expanded, ridge-like tubercle (‘pvr’ in figure 3*a*) on the cranial end of the lateral side of the cnemial crest, giving the latter structure a laterally curving appearance, although it cannot be considered ‘hooked’ [40]. A subtle, distally deeper ‘knee extensor groove’ [41] extends laterodistally to medioproximally along the lateral surface of the cnemial crest, between the cranial margin of the crest and the ridge-like tubercle, as it has been described for some ceratosaurs [42,43]. The lateral surface of the proximal part of the tibia bears longitudinally oriented structures related to the articulation of the fibula. The cranialmost of these is the lateral depression associated with the incisura tibialis, which extends for less than one fourth of the length of the bone, and tapers distally. Its smooth surface reaches the proximal margin of the bone, and caudally borders the nearly vertical ridge-like tubercle mentioned above, i.e. the ‘posteroventral ridge’ [44]. The fibular crest steeply marks the caudal margin of the incisura. Although this side is slightly damaged, it is possible to see that the fibular crest reaches the proximal end of the tibia as a well-developed ridge, as in all non-tetanuran neotheropods [45].

The tarsal articulation at the distal portion of the tibia is composed of two facets: the distal articulation *per se* and the proximally inset astragalar buttress. These are separated by a flat vertical surface that, along with the astragalar buttress, housed the ascending process of the astragalus. The distal articulation *per se* has a lateromedially elongated subtriangular distal outline, with a long-concave caudal margin, a short-straight medial margin and a long-sigmoid craniolateral margin. Together they form sharp caudolateral and craniomedial corners, and a low-angled caudomedial corner. The concavity of the caudal margin extends proximally along the tibial shaft, but seems exaggerated by taphonomic collapse of the outer bone surface. The medial portion of the craniolateral margin is concave and laterally limited by a distinct kink (‘lk’ in figure 3*d*). This corresponds to the distal expression of a ridge (‘rd’ in figure 3*e*) that extends along the vertical articulation facet for the astragalar ascending process and separates the facet for the latter from the craniolateral facet for the distal end of the fibula, as seen in other basal theropods (e.g. *Dilophosaurus wetherilli*, UCMP 77270; *Liliensternus liliensterni*, HMN MB.R. 2175; *Zupaysaurus rougieri*, UPLR 076). The portion of the tibia lateral to that corresponds to the outer malleolus, which tapers caudolaterally in distal view and has a longitudinally striated cranial surface. It is not much expanded laterally, but probably backed up part of the fibula. It has a rounded, rather than tabular [9,46] outline in cranial or caudal views and is slight distally projected, resulting in a slightly oblique angulation of the distal margin of the tibia. The astragalar buttress extends for about 80% of the mediolateral width of the distal portion of the tibia, but makes up less than one third of its craniocaudal breadth. It corresponds to a flat surface that extends straight (in cranial view) from the mediodistal corner of the bone, at an angle of about 55^°^ to the long axis of the shaft. The lateral portion of the buttress is also slightly inclined proximocaudally to distocranially, forming a subtle slot for the ascending process of the astragalus. This leads to the formation of a groove (‘lg’ in figure 3*d*) in the lateral surface of the buttress that extends proximally along the lateral surface of the tibial shaft. Finally, the distal articulation *per se* has a subtle excavation on its caudomedial corner, an incipient version of the ‘tibial notch’ [8] seen in several other theropods (e.g. *Sinraptor dongi* [47]; *Li. liliensterni*, HMN MB.R. 2175).

The partial ischium (IVIC-P-2868) is incomplete distally and also lacks most of the pubic peduncle. Only the ventral portion of the iliac articulation is preserved, which faces mainly cranially, lacking the hypertrophied antitrochanter seen in most coelophysoids (e.g. *Syntarsus rhodesiensis*, QG 1; *Coelophysis bauri*, NMMNHS 55336; *Li. liliensterni*, HMN MB.R. 2175). Between the iliac articulation and the pubic peduncle, the incised cranial margin of the bone is finished and mediolaterally convex. This implies a fully open acetabulum, where the ischium does not contribute to the inner acetabular wall. Yet, unlike from most tetanurans [47–50], there is no sign of an obturator notch between the pubic peduncle and obturator plate. Only about one fourth of the rod-like ischial shaft is preserved, the dorsal margin of which is not fully extracted from the rock. Its dorsolateral corner is continuous to a low ridge that extends towards the iliac articulation, whereas a sharper ridge (‘vr’ in figure 3*f*) extents from the ventrolateral corner of the shaft towards the dorsal margin of the pubic peduncle. The latter sets the dorsal boundary of the laminar obturator plate, and is separated from the dorsal ridge by a shallow depression on the ischial body, caudal to the acetabular incisure. Only the caudal half of the obturator plate is preserved, which has a slightly sigmoid ventral margin and is separated from the shaft by a well-developed distal notch, as typical of basal neotheropods [45].

## 6. Comparative approach

Although the holotype of *T. admirabilis* consists only of one tibia (IVIC-P-2867), the element shows numerous characters that help to clarify its phylogenetic position and testify to its taxonomic distinctiveness. In comparison with dinosaurian outgroups, basal saurischians and basal sauropodomorphs, the tibia of *T. admirabilis* shows several synapomorphies of Theropoda and Neotheropoda. A cnemial crest is synapomorphic for dinosauromorphs, and the presence of a slightly laterally curved crest, as present in *T. admirabilis*, has been found as a dinosaur synapomorphy by Nesbitt [51] and references therein, but the cnemial crest of basal saurischians, such as the herrerasaurs *Staurikosaurus pricei* (MCZ 1669) and *Herrerasaurus ischigualastensis* (PVL 2566, PVSJ 373) is craniocaudally short and accounts for one-third to two-fifths of the craniocaudal width of the proximal end. The same is true for the basal sauropodomorphs *Saturnalia tupiniquim* (MCP 3944-PV) and *Panphagia protos* (PVSJ 874) and most basal ornithischians such as *Pisanosaurus mertii* [52] and *Lesothosaurus diagnosticus* (NHMUK RUB 17). By contrast, the cnemial crest makes up half or more of the craniocaudal width of the proximal surface of the tibia in basal theropods, e.g. ‘*Sy.*’ *kayentakatae* [53], *Li. liliensterni* (MB.R. 2175), *Elaphrosaurus bambergi* (MB.R. 4960), and this is also the case in *T. admirabilis*. Several taxa that are currently considered to be amongst the most basal theropods, such as *Tawa hallae* (GR 241–242) and *Eodromaeus murphi* (PVSJ 560–562) also have a relatively short cnemial crest, indicating that *T. admirabilis* belongs to the less inclusive neotheropod clade.

As long recognized [36], theropod tibiae differ from those of other archosaurs in the presence of a ridge/crest for the attachment of the fibula on the lateral side of the proximal part of the shaft. Although a roughened patch is present in a similar position in some basal saurischians (e.g. *Eoraptor lunensis* [54]) and a sharp ridge is seen in silesaurids [55] and some basal ornithischians [56], this is only developed as a strong crest in theropods, as it is seen in *T. admirabilis*. Finally, in non-dinosaur dinosauriforms, basal saurischians and sauropodomorphs, the distal articulation of the tibia is craniocaudally wide, as is the slot that receives the ascending process of the astragalus [55,57–59]; a condition also present in *Eod. murphi* (PVSJ 560–562), *Eor. lunensis* (PVSJ 512) and *Tawa hallae* (GR 241–242). By contrast, most theropods have the facet for the ascending process of the astragalus restricted to the cranial part of a craniocaudally narrow distal articulation of the tibia. This is also seen in *T. admirabilis*, further confirming the neotheropod affinities of this taxon. Ornithischians also have a craniocaudaly narrow distal articulation of the tibia (e.g. *Le. diagnosticus*, NHMUK RUB 17; *Scutellosaurus lawleri*, MNA V175), but the slot for the ascending process of the astragalus is not as defined as in saurischians, including theropods like *T. admirabilis*.

The placement of *T. admirabilis* among neotheropods is more difficult to determine, but the type tibia shows a combination of plesiomorphic and apomorphic traits that helps to narrow down its affinities. In basal dinosauromorphs, herrerasaurs and many basal sauropodomorphs, a line connecting the distalmost points of the lateral and medial malleoli of the tibia would form an approximate right angle with the long axis of the distal part of the shaft in cranial view [52,55,57,59–61]. By contrast, in most theropods (but also in ornithischians [62,63]), the outer malleolus reaches further distally than the inner malleolus so that this line forms an oblique angle to the long axis of the shaft. In some basal taxa, such as *D. wetherilli* (UCMP V 4214) and *Gojirasaurus quayi* (MB.R. 1985), but also in *El. bambergi* (MB.R. 4997), the situation resembles that of non-theropod saurischians, and the angle is still close to a right angle in other basal forms (*Li. liliensterni* and *Z. rougieri* [8]). By contrast, the outer malleolus is further distally placed in most averostrans (*Torvosaurus tanneri* [64]; *Majungasaurus crenatissimus* [42]; *Si. dongi* [47]; see also [65]). In *T. admirabilis*, that virtual line is angled at about 80^°^, approaching the averostran condition (see the electronic supplementary material, figure S6) and indicating that the new taxon is more derived on that line than coelophysoids.

Likewise, in basal neotheropods, the astragalar buttress on the cranial side of the distal portion of the tibia is craniocaudally broad and broadens laterally, resulting in a rectangular outline of the distal end of the bone in distal view (*Z. rougieri*, UPLR 076; *Li. liliensterni*, HMN MB.R. 2175; *Sy. rhodesiensis*, QG 691, 792; *D. wetherilli*, UCMP 77270; ‘*Sy.*’*kayentakatae*[53]; ‘Petrified Forest’ coelophysoid, UCMP 128618; ‘Snyder Quarry’ theropod, NMMNHS P-29046). By contrast, in averostrans, the astragalar buttress is craniocaudally compressed and of subequal breadth throughout its width, so that the distal outline of the tibia is more triangular. The condition in *T. admirabilis* approaches the latter state, further indicating that this taxon is closer to averostrans than to coelophysoids or members of the ‘*Dilophosaurus* clade’ [9].

However, several characters indicate that *T. admirabilis* is placed outside Tetanurae and, most probably, also outside Averostra. Two different morphologies of the fibular crest are seen among theropods [45]; basal forms present a lower, more proximally placed crest connected to the proximal end of the tibia by a well-defined ridge, whereas tetanurans have a higher, more distally placed crest that arises directly out of the shaft and lacks a proximal connection. More precisely, the fibular crest of some basal tetanurans is still connected to the proximal end by a low lateral swelling [4]. In *T. admirabilis*, the fibular crest is connected to the proximal end, corresponding to the non-tetanuran condition found in basal theropods.

In addition, characters of the distal part of the tibia also indicate that *T.* *admirabilis* lies outside Averostra. In basal theropods, the astragalar buttress extends obliquely across the cranial surface of the distal portion of the tibia and flexes proximally adjacent to the lateral margin of the shaft [5,8]. In ceratosaurs [42,66,67] and basal tetanurans [47,48,64,68], this proximal flexure is placed more medially, towards the level of the mid-width of the shaft. In *T. admirabilis*, the position of this flexure, and its rather marked appearance (in contrast to a more gradual bend in tetanurans), corresponds to the condition seen in basal theropods. Furthermore, the buttress of *T. admirabilis* forms a narrow, but cranicaudally measurable platform, differing from the faint structure seen in ceratosaurs and basal tetanurans. In summary, the combination of characters exhibited by the tibia of *T. admirabilis* implies a non-averostran relationship, though probably as a sister taxon to this clade.

### 6.1 Taxonomic distinctiveness of *Tachiraptor admirabilis*

Given the fragmentary nature of the specimens referred to *T. admirabilis*, some comments on its distinction as a separate taxon might be warranted. As outlined above, the character combination exhibited by this taxon is unusual and helps to distinguish it from all other theropods described so far. Specifically, the combination of a craniocaudally narrow astragalar buttress that remains of subequal breadth throughout its width with a laterally placed bend in the buttress is unseen in any other known theropod and can thus currently be regarded as diagnostic of *T. admirabilis*. However, since this is a combination of derived (excluding it from Coelophysoidea and other basal theropods) and plesiomorphic (excluding it from Averostra) characters, future finds might demonstrate that such a combination may not be unique for this taxon, but characterize close averostran outgroups.

However, in addition to this unique character combination, *T.* *admirabilis* also differs from other early theropods in the morphology of the fibular condyle, which extends slightly more caudally than the medial condyle and forms a sharp angle. In theropod outgroups, such as *Eor. lunensis* [54], *Sa. tupiniquim* [59], *P. protos* [PVSJ 874] and herrerasaurs [57,60], the caudolateral corner of the fibular condyle is rounded and does not reach as far caudally as the medial condyle. Although the fibular condyle is more caudally extensive in several basal theropods (e.g. *D.* *wetherilli*, UCMP V 4214 [45], *Li.* *liliensterni*, MBR 2175, *Masiakasaurus* *knopfleri* [41]), its caudolateral corner is rounded. This is also the case in more derived theropods [9,42,48,64], in which the fibular condyle ends well cranial to the caudal margin of the medial condyle. Thus, the shape and extent of the fibular condyle may be autapomorphic for *T. admirabilis*.

**Figure 4.**
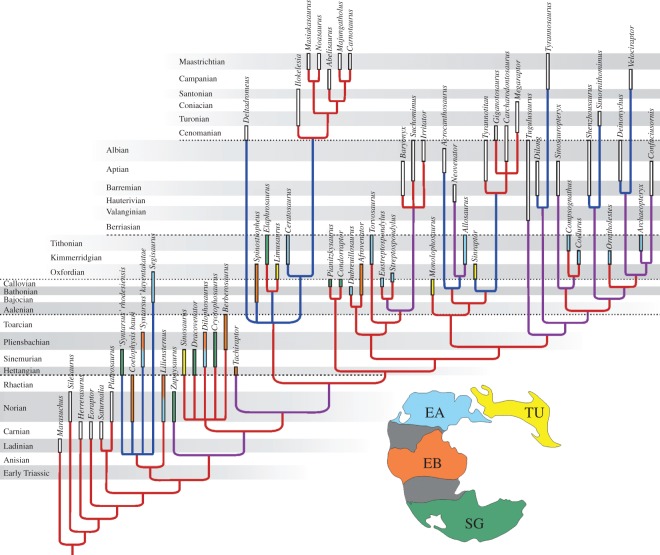
Strict consensus of the 1107 MPTs recovered with the inclusion of *Tachiraptor admirabilis* into the dataset of Xu *et al.* [12]. Branch colours represent extension of ghost lineages in millions of years (red, less than 15; purple, 15–35; blue, more than 35). Taxon bar lengths correspond to their chronologic distribution/uncertainty (based on various sources). Bar colours match those of the index Middle Jurassic palaeomap [70] and correspond to the provenance of Triassic/Jurassic theropods from the defined palaeobiogeographic provinces (SG, South Gondwana; EA, Euramerica; TU, Transurals; EB, Equatorial Belt) at the time of their occurrences.

### 6.2 Affinities of *Tachiraptor admirabilis*

To explore the phylogenetic placement of *T. admirabilis* within Theropoda, the new taxon was scored into the taxon/character matrix of Xu *et al.* [12]. This is mainly based on the original dataset of Smith *et al.* [9], which is the most recent cladistic study to comprehensively sample the evolutionary segment (basal Neotheropoda) into which *T. admirabilis* seems to belong. The parsimony analysis was performed using TNT [69], employing the same parameters for heuristic searches (number of replicates, ordering strategies) as Xu *et al.* [12], but excluding its controversial 413th character. A total of 21 characters were scored for the holotype tibia and associated ischium of *T. admirabilis*, as well as for the astragalar anatomy inferred from tibial traits. In total, this corresponds to slightly more than 5% of the characters in the dataset. A total of 1107 most parsimonious trees (MPTs), with lengths of 1144 steps, were recovered. Their strict consensus (figure 4) agrees with the results of Xu *et al.* [12] in all aspects, and recovers *T. admirabilis* as the sister taxon to Averostra. The same position is found if *T. admirabilis* is scored in the original dataset of Smith *et al.* [9]. Additionally, if the data matrix of Smith *et al.* is modified as suggested by Brusatte *et al.* [71], the strict consensus of 56 MPTs shows *T. admirabilis* forming a large polytomy together with *Cryolophosaurus ellioti*, *D. wetherilli*, *Dracovenator regenti*, *Sinosaurus triassicus*, *Z. rougieri* and Averostra. Details of the phylogenetic analyses can be found in the electronic supplementary material.

## 7. Discussion: biochronology and palaeobiogeography

Averostra is the main clade of theropods and includes all known post-Early Jurassic forms, but its origin is still poorly understood. Unambiguous records of Averostra, i.e. Tetanurae and Ceratosauria, are no older than Middle Jurassic [3,4], but recent data from central Patagonia constrains the major diversifications of Tetanurae and Ceratosauria to as early as the Toarcian-Aalenian (Early–Middle Jurassic) boundary [72]. A number of older taxa have variously been regarded as early averostrans, but their phylogenetic positions are disputed. Possibly the best candidate for an Early Jurassic averostran is *C. ellioti* from Antarctica [9,73]. Sometimes considered an early allosauroid [74], it has recently been recovered as a basal tetanuran, outside the megalosauroid-neotetanuran dichotomy [4]. However, other analyses found this taxon as part of an informally named ‘*Dilophosaurus* clade’ outside Averostra [7,9,12,75]. The same is the case for *Sinosaurus triassicus* (i.e. ‘*Dilophosaurus*’ *sinensis*), which has variably been regarded as a basal member of the Tetanurae [4] or the ‘*Dilophosaurus* clade’ [7,9,12]. Finally, *Berberosaurus liassicus* from the Pliensbachian-Toarcian of Morocco was originally proposed to be an abelisauroid [10], and later found to be a more basal ceratosaur [2,3]. However, Xu *et al.* [12] found this taxon outside Averostra, also as part of the ‘*Dilophosaurus* clade’.

The detailed phylogenetic arrangement of basal theropods has implications for our understanding of the timing of Averostra origins. The placement of several Early Jurassic taxa in the ‘*Dilophosaurus* clade’ [7,9,12,75] implies a minimal ghost lineage of 25 Myr, with the origin of Averostra in the Hettangian at the latest. On the contrary, the hypothesis of Carrano *et al.* [4], with the Hettangian-Sinemurian *Sinosaurus* *triassicus* as the oldest averostran and the latter clade forming the sister group of Coelophysoidea, which also includes *D. wetherilli*, implies a minimal ghost lineage of nearly 30 Myr and a Norian minimal age for the origin of Averostra. It is worth mentioning, however, that the analysis of Carrano *et al.* [4] focuses on tetanuran interrelationships and has a quite limited sampling of more basal taxa.

The discovery of *T. admirabilis* and its phylogenetic placement as the sister taxon of Averostra would considerably reduce the ghost lineage for the latter clade under the phylogenetic hypothesis of Carrano *et al.* [4], as it places the minimal age for the origin of this group in the Hettangian, matching the probable age for *Sinosaurus triassicus* [76]. On the contrary, under the phylogenetic hypothesis of Nesbitt *et al.* [11,77,78], the inferred age and phylogenetic position of *T. admirabilis* would enlarge the stratigraphic gap at the base of Averostra. In particular, under an inclusive ‘*Dilophosaurus* clade’ hypothesis, as favoured by our analysis, an equally large (nearly 30 Myr) ghost lineage would be recovered for Averostra (figure 4). Indeed, in the phylogenetic hypothesis presented here, a longer (more than 35 Myr) ghost lineage is seen only for Abelisauroidea, as the other cases (marked in blue in figure 4) are obvious biases caused by the small sample size of the corresponding groups (tyrannosaurs, ornithomimosaurs and dromaeosaurs) or result from ambiguous (e.g. coelophysoids) or controversial (e.g. *Acrocanthosaurus*, *Deltadromeus*, *Sinraptor* [4,79]) positioning of taxa. Still, the Abelisauroidea ghost lineage has been ‘shifted’ to the less inclusive abelisaurid and noasaurid subgroups by the discovery of *Eoabelisaurus mefi* from the Middle Jurassic of Argentina [3]. Indeed, depending on the chosen phylogenetic hypothesis, the greatest stratigraphic gap in the Jurassic record of Theropoda appears to be that at the stem of the Averostra.

The peculiar provenance of *T. admirabilis*, coupled with the long ghost range of its sister group, justifies a survey for background palaeobiogeographic patterns, as the stratigraphic gap of that lineage may be in part explained by the meagre palaeontological sampling from certain Gondwanan areas [13]. Yet, a straightforward Laurasia-Gondwana dichotomy, as well as views based on modern day continental partitioning, were challenged by alternative approaches that integrate data from past climate and biome distributions [3,80]. In our case, several lines of evidence suggest that northern South America was more closely associated with the Laurasian realm (e.g. southern North America) than to southern Gondwana [70,81]. Indeed, Rees *et al.* [70] suggest that, together with North Africa, the former areas were part of a summer-wet equatorial belt bordered to the north and south by extensive deserts. Accordingly, in order to further investigate the palaeobiogeographic patterns of Jurassic theropod faunas, we identify four biogeographic provinces based on Triassic–Jurassic palaeogeographic reconstructions [70,82]. These include an Equatorial Belt separated by desert belts from South Gondwana and a northern landmass, which is further divided longitudinally into Euramerican and Trans-Uralian domains (figure 4). This latter separation is more pronounced from the Middle Jurassic onwards, with the expansion of the Turgai seaway [83], but East Asia (which hosts most of the Asian theropod records of Jurassic age) was hitherto separated from western parts of Laurasia by the Mongol-Okhotsk Ocean [82,84].

Because of the major uncertainties regarding basal theropod evolution, we decided not to employ the resulting topology of our analysis (which aimed to place *T. admirabilis* in a phylogenetic context, not to solve basal theropod relationships) to conduct a dispersal–vicariance analysis [85,86] using the software RASP [87]. Instead, we assembled an informal supertree of basal theropods, with the framework of our reanalysis of Xu *et al.* [12], with the addition of phylogenetic hypotheses for Coelophysoidea [8], Ceratosauria [3] and Tetanurae [4], slightly modified to produce a fully resolved tree (see the electronic supplementary material figure S4). Only Triassic–Jurassic neotheropods were included, with younger sister taxa represented by terminal branches. Ancestral geographical ranges were then mapped under those topological constrains, with the biogeographic events (i.e. expansion of range/dispersal) restricted to adjacent areas, i.e. between Euramerica and the Equatorial Belt, and between those areas and, respectively, the Transurals and South Gondwana. Our results (see the electronic supplementary material) resolve the ancestral range for Tetanurae as the Transurals plus Euramerica or the former area alone (with prevalence of this latter reconstruction). For Ceratosauria, the ancestral range is more southern: Equatorial Belt or that area plus South Gondwana. Down the tree, the ancestral range for Averostra is either Transuralian or a more western area including Euramerica plus the Equatorial Belt (with most reconstructions supporting the first option). The record of *T. admirabilis* in the latter area indicates a more southern ancestral range for the clade it forms with Averostra, i.e. the Equatorial Belt or that area plus South Gondwana.

The above results highlight the pivotal role of the Equatorial Belt in the Jurassic biogeographic events that shaped theropod evolution. Indeed, the worldwide distribution of most non-averostran groups in the Early Jurassic [6,88] shows that there was ample space for a rapid dispersal/extension of geographical ranges at that time. In this context, the more northern ancestral range of Tetanurae and more southern ancestral range of Ceratosauria, rather intuitively hints at an intermediary ‘connection’ range for stem-averostrans, into which the record *T. admirabilis* straightforwardly fits. In the end, the ancestral range of Averostra suggests that the stratigraphic gap at its base is more likely to be filled by new Early Jurassic records from the northern continents [38,88,89] or north Gondwana [90] than from more southern areas [7,73] of that supercontinent.

## Supplementary Material

Electronic Supplementary Material for: New dinosaur (Theropoda, stem-Averostra) from the earliest Jurassic of the La Quinta Formation, Venezuelan Andes The file includes: 1. Details of the U-Pb Geochronology analysis. 2. Institutional Abbreviations. 3. Details of the phylogenetic analyses. 3.1. Scoring of Tachiraptor admirabilis in the data set of Xu et al. [12]. 3.2. Reanalysis of Smith et al. [9] including Tachiraptor admirabilis. 3.3. Reanalysis of Smith et al. [9] as modified by Brusatte et al. [70] including Tachiraptor admirabilis. 4. Details of the palaeobiogeography analysis. 5. Further comparison of the distal tibia articulation of Tachiraptor admirabilis.
