# Addendum to ‘New dinosaur (Theropoda, *stem*-Averostra) from the earliest Jurassic of the La Quinta formation, Venezuelan Andes’

**DOI:** 10.1098/rsos.140527

**Published:** 2014-12-24

**Authors:** Max C. Langer, Ascanio D. Rincón, Jahandar Ramezani, Andrés Solórzano, Oliver W. M. Rauhut

**Affiliations:** 1 Laboratório de Paleontologia de Ribeirão Preto, FFCLRP, Universidade de São Paulo, Avenida Bandeirantes 3900, 14040-901 Ribeirão Preto-SP, Brazil; 2 Laboratorio de Paleontología, Centro de Ecología, Instituto Venezolano de Investigaciones Científcas (IVIC), Carretera Panamericana Km 11, 1020-A Caracas, Venezuela; 3 Department of Earth, Atmospheric and Planetary Sciences, Massachusetts Institute of Technology, 77 Massachusetts Avenue, Cambridge, MA, USA; 4 SNSB, Bayerische Staatssammlung für Paläontologie und Geologie and Department of Earth and Environmental Sciences, Ludwig-Maximilians-University, Richard-Wagner-Strasse 10, Munich, Germany

This addendum is to ensure that the ICZN (International Code of Zoological Nomenclature) criteria for the availability of new names are satisfied. This addendum, being a ‘corrigendum’ in the sense of the Glossary of the Code, should be consulted alongside the original publication [1] as only both together fulfil the ICZN criteria. The date of publication of the nomenclatural acts is the date that this addendum has been published.

The holotype of *Tachiraptor admirabilis* is IVIC-P-2867 and is housed at Colección Paleontológica del Centro de Ecología, Instituto Venezolano de Investigaciones Científicas, Caracas, Venezuela. The full name and location of the repository is listed in the electronic supplementary material of the original article, but should have been included in the main article text.

The published work and the nomenclatural acts it contains (new genus and species names) have also been registered in Zoobank under the following LSIDs (Life Science Identifiers):

Original Publication: 8951A1E3-E9EB-4DB9-808D-2DB424686941

This addendum: F8786B0B-FDCB-4A7C-AD5B-EAAD65E818F0

Genus: C9F4BDB3-D0C8-4F37-A485-820BC0BABDEE

Species: 65710920-94AF-4778-8412-434868D5F214

This information was not included in the original paper.
