# Supplementary material for: New dinosaur (Theropoda, stem-Averostra) from the earliest Jurassic of the La Quinta formation, Venezuelan Andes
Source: R Soc Open Sci. 2014 Oct 8;1(2):140184. doi: 10.1098/rsos.140184 (PMC4448901; doi:10.1098/rsos.140184)
Supplement: Electronic Supplementary Material for: New dinosaur (Theropoda, stem-Averostra) from the earliest Jurassic of the La Quinta Formation, Venezuelan Andes The file includes: 1. Details of the U-Pb Geochronology analysis. 2. Institutional Abbreviations. 3. Details of the phylogenetic analyses. 3.1. Scor [file rsos140184supp1.docx]

Electronic Supplementary Material for:

New dinosaur (Theropoda, stem-Averostra) from the earliest Jurassic of the La Quinta Formation, Venezuelan Andes

**Max C. Langer^1,^*, Ascanio D. Rincón^2^, Jahandar Ramezani ^3^,Andrés Solórzano^2^**

**and Oliver W. M. Rauhut^4^**

^1^ *Laboratório de Paleontologia de Ribeirão Preto, FFCLRP, Universidade de São Paulo, Av. Bandeirantes 3900, 14040-901, Ribeirão Preto-SP, Brazil*.

^2^ *Laboratorio de Paleontología, Centro de Ecología, Instituto Venezolano de Investigaciones Científcas (IVIC), Carretera Panamericana Km 11, 1020-A, Caracas, Venezuela*.

^3^ *Department of Earth, Atmospheric and Planetary Sciences, Massachusetts Institute of Technology, 77 Massachusetts Ave., Cambridge, MA, USA*

^4^ *SNSB, Bayerische Staatssammlung für Paläontologie und Geologie and Department of Earth and Environmental Sciences, Ludwig-Maximilians-University, Richard-Wagner-Str. 10, Munich, Germany*.

**This file includes:**

1. Details of the U-Pb Geochronology analysis.

2. Institutional Abbreviations.

3. Details of the phylogenetic analyses.

3.1. Scoring of *Tachiraptor admirabilis* in the data set of Xu et al. [12].

3.2. Reanalysis of Smith et al. [9] including *Tachiraptor admirabilis*.

3.3. Reanalysis of Smith et al. [9] as modified by Brusatte et al. [70] including *Tachiraptor admirabilis*.

4. Details of the palaeobiogeography analysis.

5. Further comparison of the distal tibia articulation of *Tachiraptor admirabilis*.

**1. Details of the U-Pb Geochronology analysis.**

| 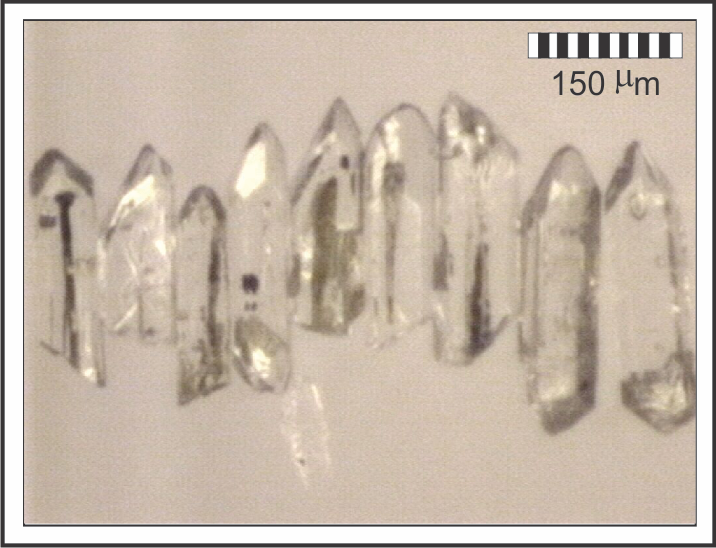 | 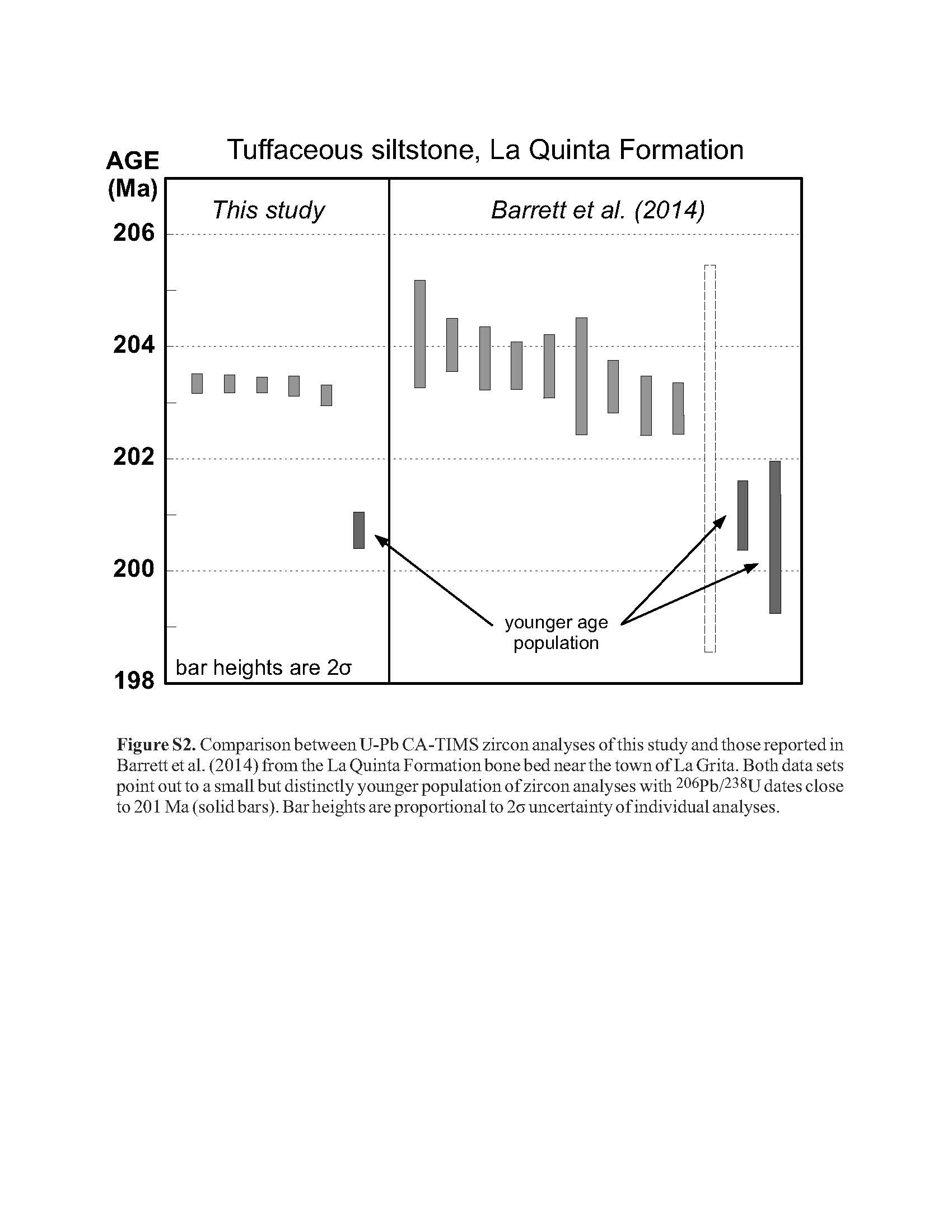 |
| --- | --- |

**Figure S1**. Analyzed zircon crystals and comparison between U-Pb CA-TIMS zircon analyses of this study and those reported in Barrett et al. [20] from the La Quinta Formation bone bed near the town of La Grita. Both data sets point out to a small but distinctly younger population of zircon analyses with 206Pb/238U dates close to 201 Ma (solid bars). Bar heights are proportional to 2σ uncertainty of individual analyses.

**2. Institutional Abbreviations.**

**BMNH,** Natural History Museum, London, UK; **GR**, Ruth Hall Museum of Paleontology at Ghost Ranch, USA; **HMN**, Museum für Naturkunde der Humboldt Universität, Berlin, Germany; **IVIC**, Colección Paleontológica del Centro de Ecología, Instituto Venezolano de Investigaciones Científicas, Caracas, Venezuela; **MCP**, Museu de Ciências e Tecnologia PUC/RS, Porto Alegre, Brazil; **MCZ**, Museum of Comparative Zoology, Harvard University, Cambridge, USA; **NMMNHS**, New Mexico Museum of Natural History and Science, Albuquerque, USA; **MNA**, Museum of Northern Arizona, Flagstaff, USA; **PVL**, Instituto Miguel Lillo, Tucumán, Argentina; **PVSJ**, Museo de Ciencias Naturales de la Universidad Nacional de San Juan, San Juan, Argentina; **QG**, National Museum of Natural History, Bulawayo, Zimbabwe; **UCMP**, University of California Museum of Paleontology, Berkeley, USA; **UPLR**, Museo de Paleontologia, Universidad Nacional de La Rioja, La Rioja, Argentina.

**3. Details of the phylogenetic analyses.**

3.1. Scoring of *Tachiraptor admirabilis* in the data set of Xu et al. [12].

| ?????????????????????????????????????????????????? ??????????????????????????????????????????????????  ?????????????????????????????????????????????????? ??????????????????????????????????????????????????  ?????????????????????????????????????????????????? ???????????????????????????????????0001???????????  ????001110110??????01?1??????????????????????????? ??????????????????????????????????????????????????  ??????0????? |
| --- |

3.2. Reanalysis of Smith et al. [9] including *Tachiraptor admirabilis.*

*Tachiraptor admirabilis* was scored into the original taxon/character matrix of Smith et al. [9], which is the main basis for the study of Xu et al. [12]. The parsimony analysis was performed using TNT [69], employing the same parameters for heuristic searches (number of replicates, weighing/ordering strategies) of the original study. A total of twenty character states were scored from a total of 347 (which correspond to the first 347 of the matrix provided above). Eight most parsimonious trees (MPTs), 833 steps long were recovered after a search with random seed = 0 and hold = 20. Their strict consensus (Fig. S2) agrees with the original result in all aspects, with the placement of *T. admirabilis* as the sister taxon to Averostra.


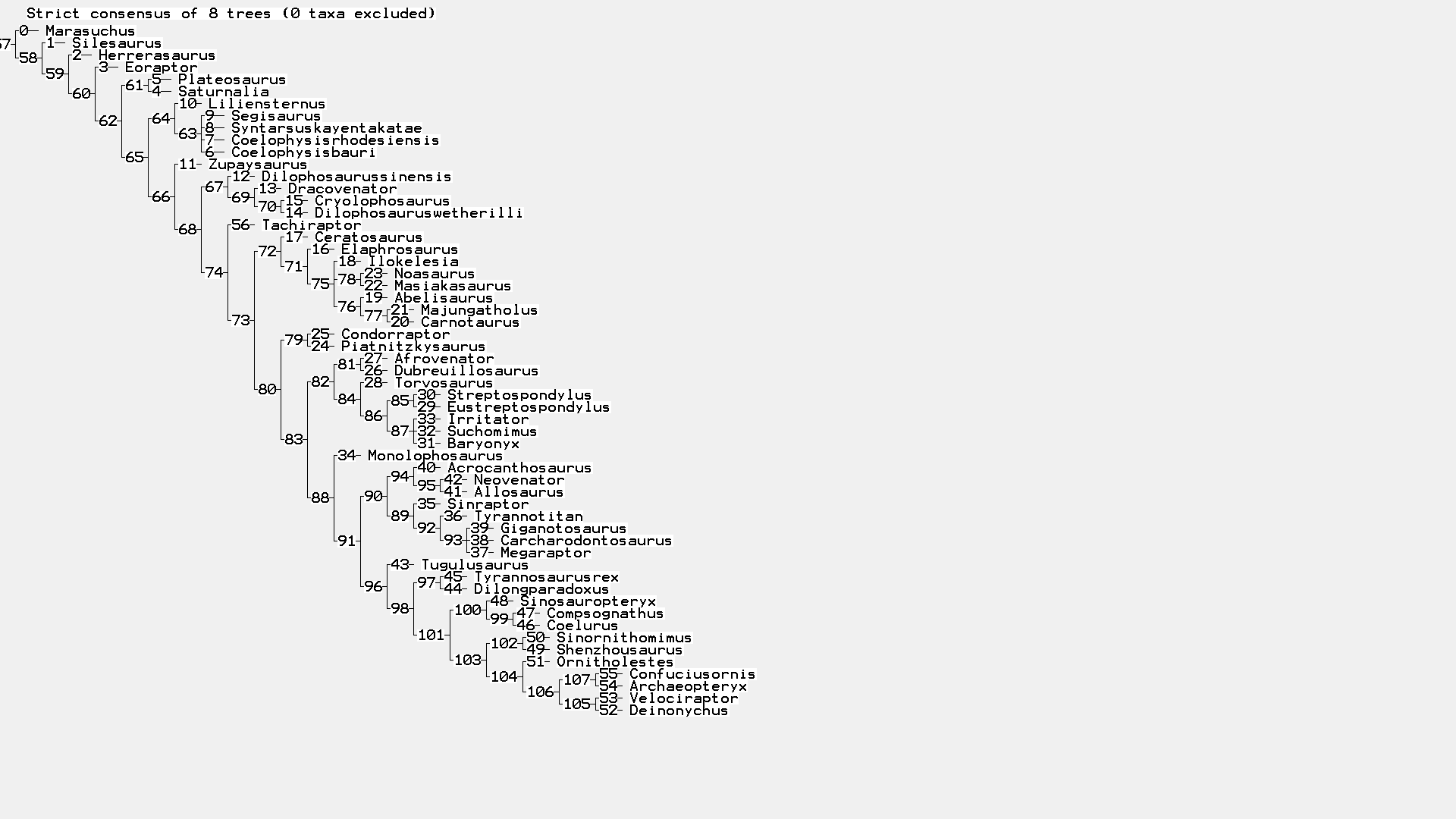


**Figure S2**. TNT output showing the strict consensus of the 8 MPTs found with the inclusion of *Tachiraptor admirabilis* in the data-set of [9].

3.3. Reanalysis of Smith et al. [9] as modified by Brusatte et al. [70] including *Tachiraptor admirabilis*

The inclusion of *Tachiraptor admirabilis* in the data matrix of Smith et al. [9], as modified by Brusatte et al. [70] led to 56 MPTs (831 steps long) the strict consensus of which places *T. admirabilis* in a large polytomy together with *Cryolophosaurus ellioti*, *Dilophosaurus wetherilli*, *Dracovenator regenti*, *Sinosaurus triassicus*, *Zupaysaurus rougieri*, and Averostra. A 50% majority-rule consensus tree (Fig. S3) resolves *Z. rougieri* as more basal and *T. admirabilis* + *S. triassicus* as closer to Averostra.

**
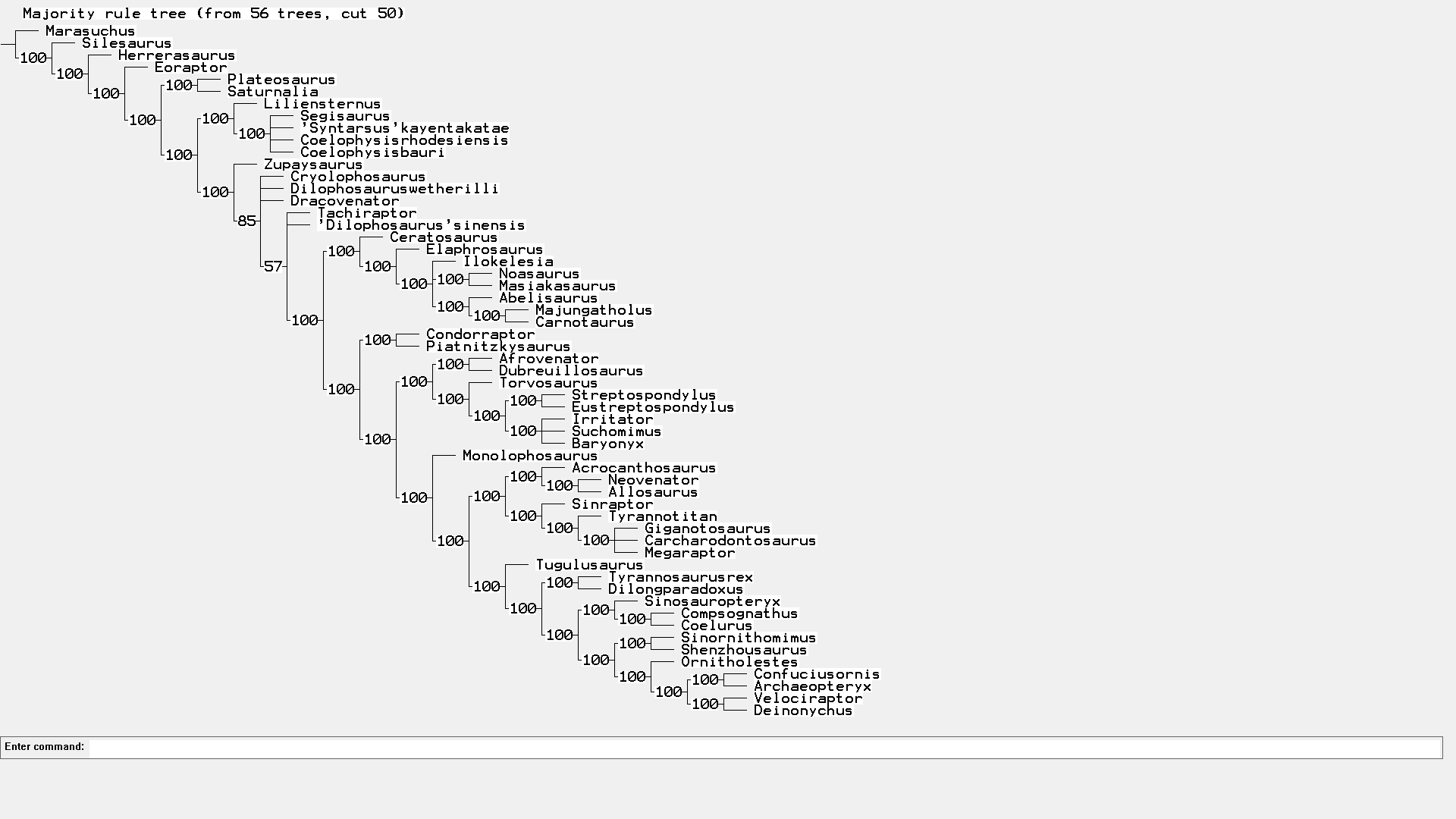
**

**Figure S3**. TNT output showing the 50% majority rule consensus of the MPTs found with the inclusion of *Tachiraptor admirabilis* in the data-set of Smith et al. [9] as modified by Brusatte et al. [70].

**4. Details of the palaeobiogeography analysis.**

The Dispersion-Vicariance Analysis [85–86] mapped ancestral geographic ranges over a topology framework based on our reanalysis of Smith et al. [9], added of more compressive phylogenies of Coelophysoidea [8], Tetanurae [4] and Ceratosauria [3], slightly modified to produce a fully resolved tree (figure S3). Only Triassic-Jurassic neotheropods were included, with their younger sister clades represented by terminal branches.

| 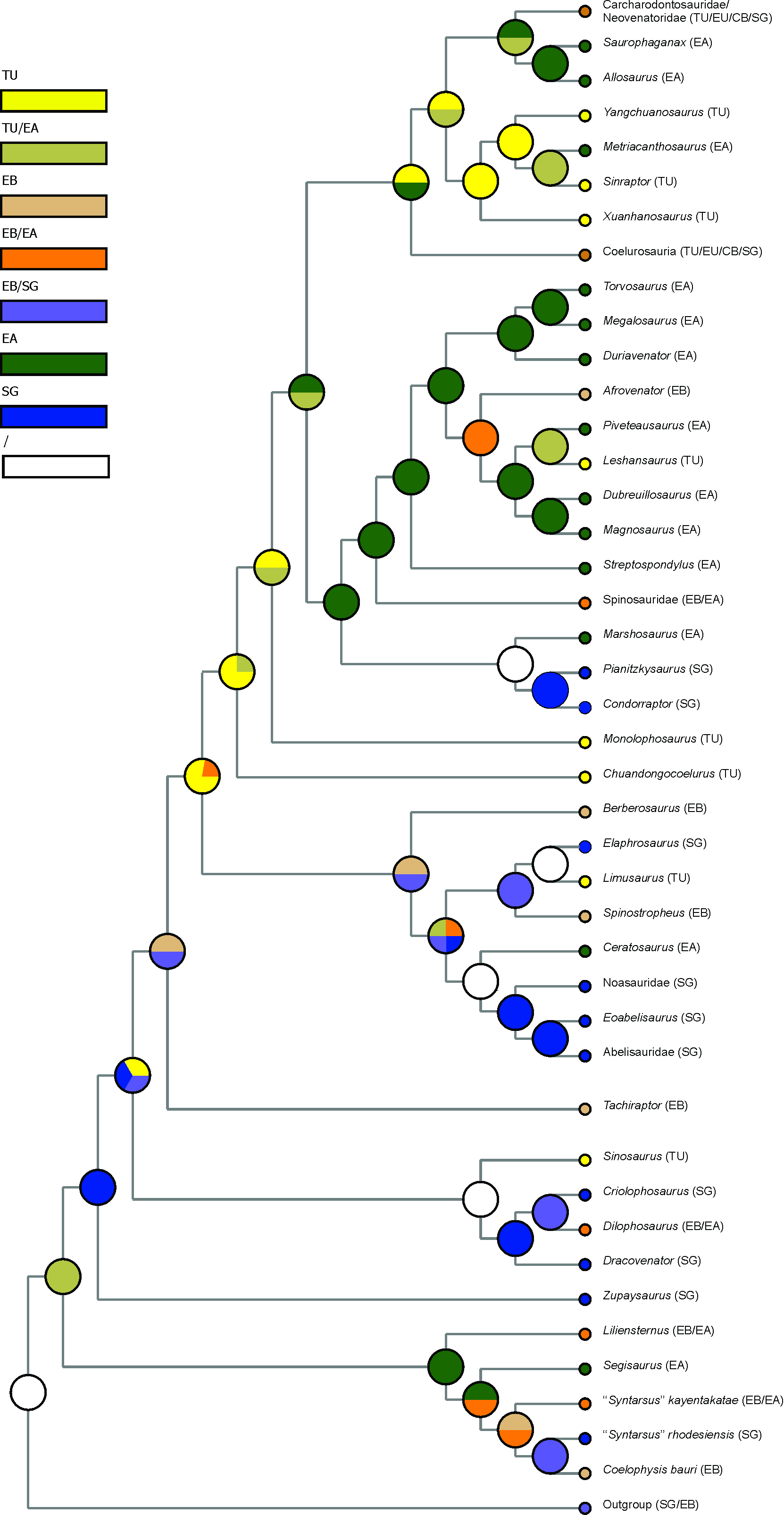 | **Figure S4**. DIVA analysis showing ancentral ranges for early rise of theropods. Paleobiogeographical provinces codes as follows: SG = South Gondwana; EB = Equatorial Belt; EA = Euramerica; TU = Transurals. Color index does not follow that of figure S5. |
| --- | --- |

Figure S5 depicts the provenance of each taxon plotted in figure S4. These are based on the mapping of the respective fossil bearing strata into the presumed range of the palaeobiogeographical provinces at the time they were deposited. Late Triassic taxa (underlined) are plotted in the Early Jurassic map.


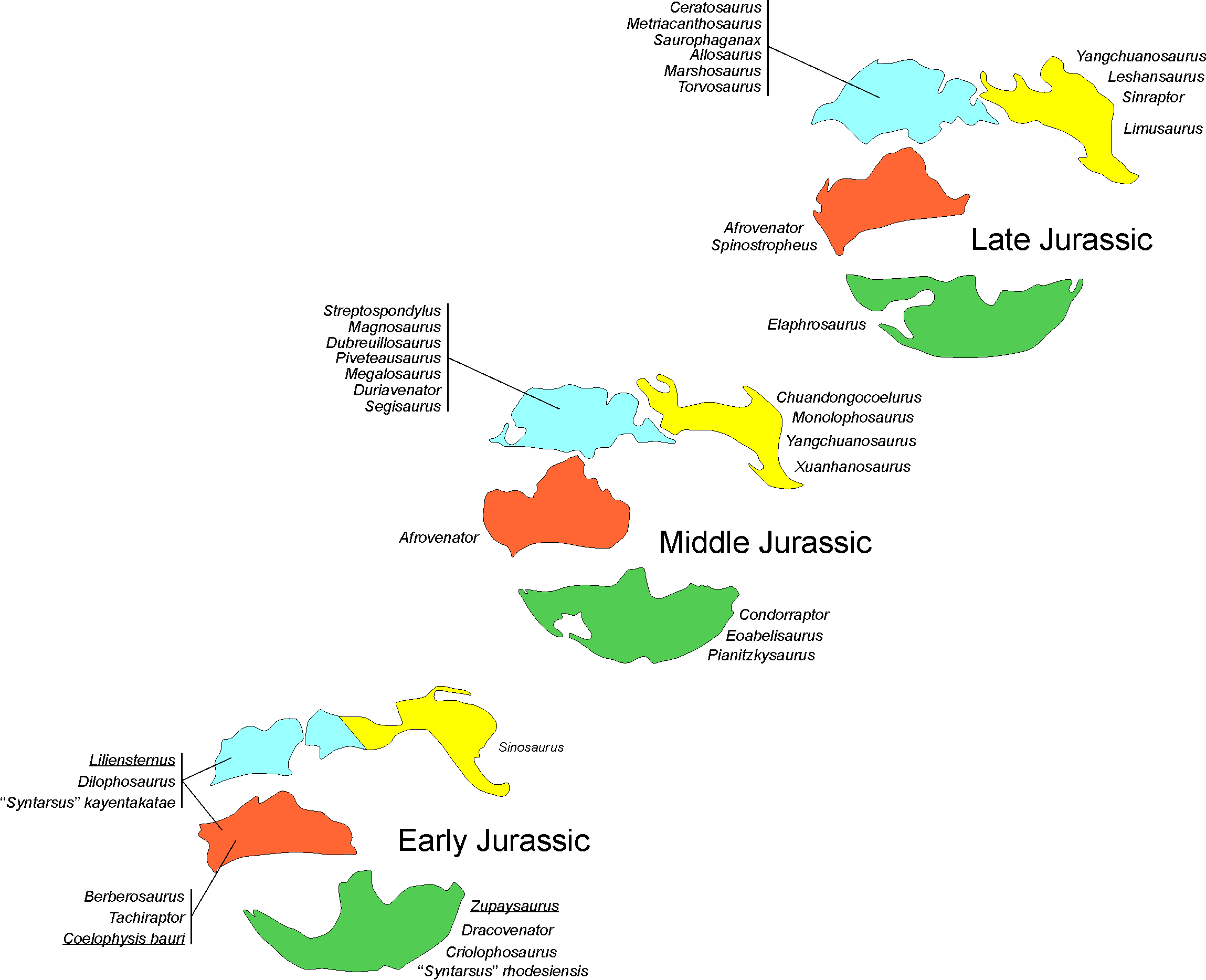


**Figure S5**. Early to Late Jurassic paleomaps based on [80], with provenance of theropod taxa.

**5. Further comparison of the distal tibia articulation of *Tachiraptor admirabilis*.**

**
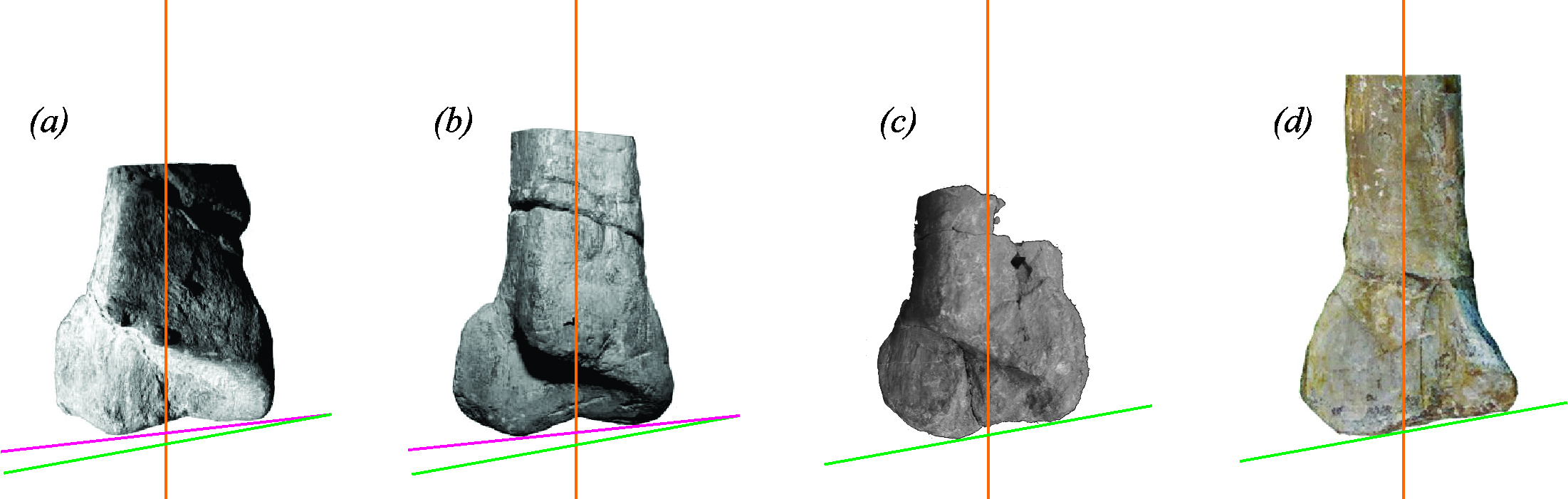
**

**Figure S6**. Distal portion of the tibia of the basal theropods (a) *Zupaysaurus rougieri* [8]; (b) *Liliensternus liliensterni* [8], (c) *Tachiraptor admirabilis* (IVIC-P-2867), and (d) *Chuandongocoelurus primitivus* [65] in cranial view showing the distal displacement of the outer malleolus. Orange line = long axis of the tibial shaft; green/pink lines = oblique lines across the distalmost projections of the outer and inner malleoli; Green lines form 80° angles to the long axis of the shaft (approximate condition of *T. admirabilis* and *C. primitivus*) and pink lines form 85° angles to the long axis of the shaft (approximate condition of *Z. rougieri* and *L. liliensterni*).
